# HDAC Inhibition Counteracts Metastatic Re-Activation of Prostate Cancer Cells Induced by Chronic mTOR Suppression

**DOI:** 10.3390/cells7090129

**Published:** 2018-09-01

**Authors:** Jasmina Makarević, Jochen Rutz, Eva Juengel, Sebastian Maxeiner, Jens Mani, Stefan Vallo, Igor Tsaur, Frederik Roos, Felix K.-H. Chun, Roman A. Blaheta

**Affiliations:** 1 Department of Urology, Goethe-Universität Frankfurt, D-60590 Frankfurt am Main, Germany; jmakarevic@air-net.de (J.M.); Jochen.Rutz@kgu.de (J.R.); Eva.Juengel@unimedizin-mainz.de (E.J.); SebastianMaxeiner@gmx.de (S.M.); Jens.Mani@kgu.de (J.M.); Stefan.Vallo@kgu.de (S.V.); Igor.Tsaur@unimedizin-mainz.de (I.T.); Frederik.Roos@kgu.de (F.R.); Felix.Chun@kgu.de (F.K.-H.C.); 2 Department of Urology and Pediatric Urology, University Medical Center Mainz, D-55131 Mainz, Germany

**Keywords:** mTOR, histone deacetylase, prostate cancer, integrins, adhesion, invasion

## Abstract

This study was designed to investigate whether epigenetic modulation by histone deacetylase (HDAC) inhibition might circumvent resistance towards the mechanistic target of rapamycin (mTOR) inhibitor temsirolimus in a prostate cancer cell model. Parental (par) and temsirolimus-resistant (res) PC3 prostate cancer cells were exposed to the HDAC inhibitor valproic acid (VPA), and tumor cell adhesion, chemotaxis, migration, and invasion were evaluated. Temsirolimus resistance was characterized by reduced binding of PC3^res^ cells to endothelium, immobilized collagen, and fibronectin, but increased adhesion to laminin, as compared to the parental cells. Chemotaxis, migration, and invasion of PC3^res^ cells were enhanced following temsirolimus re-treatment. Integrin α and β receptors were significantly altered in PC3^res^ compared to PC3^par^ cells. VPA significantly counteracted temsirolimus resistance by down-regulating tumor cell–matrix interaction, chemotaxis, and migration. Evaluation of integrin expression in the presence of VPA revealed a significant down-regulation of integrin α5 in PC3^res^ cells. Blocking studies demonstrated a close association between α5 expression on PC3^res^ and chemotaxis. In this in vitro model, temsirolimus resistance drove prostate cancer cells to become highly motile, while HDAC inhibition reversed the metastatic activity. The VPA-induced inhibition of metastatic activity was accompanied by a lowered integrin α5 surface level on the tumor cells.

## 1. Introduction

Prostate cancer (PCa) remains a leading cause of death in men worldwide [1]. Once metastasized, PCa is difficult to treat and though androgen suppression prolongs survival it is not curative. In the last decade, several promising cytotoxic and immunological agents, bone-seeking radionuclides, and next-generation androgen receptor axis targeted compounds have been introduced to optimize treatment [2]. Still, no matter which drug is employed, resistance develops over time, leading to aggressive tumor re-growth and disappointing survival rates. To improve patient outcome, innovative and novel treatment concepts are needed, with targeted therapies increasingly gaining importance.

Due to the importance of the mechanistic target of rapamycin (mTOR) pathway in tumor development and progression, mTOR inhibitors have been designed or are under development for cancer treatment [3]. The mTOR inhibitors everolimus and temsirolimus have already been approved for the treatment of metastatic renal cell carcinoma (temsirolimus, everolimus), mantle cell lymphoma (temsirolimus), breast cancer (everolimus), and pancreatic neuroendocrine tumors (everolimus) [4].

Ciccarese et al. recently reported that about a quarter of cases of localized PCa display activating mutations of the mTOR pathway. In castration-resistant PCa, the mTOR pathway was most frequently mutated [5]. A tissue microarray study has shown that mTOR is up-regulated in PCa of all stages and grades [6]. It is thought, therefore, that targeting this pathway could lead to improved patient survival and therapeutic efficacy [7].

Nevertheless, it must be emphasized that treatment with mTOR inhibitors alone may not exert long-lasting anti-tumor effects. Rather, chronic use of either temsirolimus or everolimus in vitro has been associated with negative feedback loops leading to mitotic progression and accelerated cell proliferation [8,9]. In a clinical trial, mono-treatment with temsirolimus was not as efficient in castration-resistant PCa patients as had been expected. The trial was therefore prematurely stopped [10]. Earlier studies, have shown that epigenetic modulation by a histone deacetylase (HDAC) inhibitor might improve an mTOR inhibitor-based regime. Indeed, a single molecule inhibitor targeting both HDAC activity and Akt–mTOR signaling has recently been developed. Greater tumor growth inhibition and pro-apoptotic activity have been observed than when employing single-target Akt/mTOR or HDAC inhibitors in vitro and in vivo [11].

Combining an mTOR inhibitor with an HDAC inhibitor has been shown to exert synergistic effects on B-cell acute lymphoblastic leukemia, compared to isolated drug treatment [12]. Other groups have also demonstrated that treating prostate cancer cells with this drug combination has a synergistic blocking effect on tumor proliferation [13]. A phase I study on patients with renal cell carcinoma revealed beneficial effects of a combined mTOR–HDAC inhibitor protocol [14]. Counteracting drug resistance encountered when treating different cancers by means of pharmacologic interaction with epigenetic machinery has been shown possible in vitro, in vivo, and in clinical studies [9,15,16,17]. The present study was therefore designed to evaluate the effects of HDAC inhibition on the metastatic and invasive behavior of temsirolimus (TEM)-resistant prostate cancer cells.

## 2. Materials and Methods

### 2.1. Cell Culture

The human prostate tumor cell line PC3 was obtained from DSMZ (Braunschweig, Germany). The tumor cells were grown and subcultured in RPMI 1640 medium (Gibco/Invitrogen, Karlsruhe, Germany) supplemented with 10% fetal calf serum (FCS), 2% HEPES (2-[4-(2-hydroxyethyl)piperazin-1-yl]ethanesulfonic acid) buffer (1 M, pH 7.4), 2% glutamine, and 1% penicillin/streptomycin at 37 °C in a humidified, 5% CO_2_ incubator. Temsirolimus-resistant sublines were developed over 12 months of continuous exposure to temsirolimus (Torisel^®^, LC Laboratories, Woburn, MA, USA), starting at 1 nm/mL and increasing stepwise to 10 µm/mL (PC3^res^). Control cells remained untreated (PC3^par^). The cell doubling times of 22.07 h (PC3^par^) and 24.05 h (PC3^res^) were calculated according to the following formula:

$$\mathrm{Duration}\;\mathrm{of}\;\mathrm{culture}=\frac{\ln\left(2\right)}{\ln\left(\mathrm{final}\;\mathrm{cell}\;\mathrm{number}\right)-\ln\left(\mathrm{initial}\;\mathrm{cell}\;\mathrm{number}\right)}$$

Human endothelial cells (human umbilical vein endothelial cells; HUVECs) were isolated from human umbilical veins and harvested by enzymatic treatment with dispase (Gibco/Invitrogen). They were grown in Medium 199 (M199; Biozol, Munich, Germany), supplemented with 10% FCS, 10% pooled human serum, 20 µg/mL endothelial cell growth factor (Boehringer, Mannheim, Germany), 0.1% heparin, 100 ng/ml gentamycin, and 20 mm HEPES buffer (pH 7.4). Subcultures from passages 2–6 were selected for experimental use.

### 2.2. Drugs

Temsirolimus (TEM) was dissolved in dimethylsulfoxide (DMSO) as a 10-mm stock solution and stored as aliquots at −20 °C. Prior to experiments, TEM was diluted in cell culture medium to a final concentration of 10 µm/mL. Control cell cultures received cell culture medium without TEM. This treatment procedure lasted for at least 1 year. To exclude toxic effects of the compounds, cell viability was determined by trypan blue (Gibco/Invitrogen, Karlsruhe, Germany). Valproic acid (VPA; G.L. Pharma GmbH, Lannach, Austria), which served as a HDAC inhibitor prototype, was used at a final concentration of 1 mm. PC3^par^ or PC3^res^ cells were pre-treated for 3 days with VPA before adhesion and chemotaxis experiments were performed.

The response to therapeutic TEM concentrations (drug re-treatment) was also investigated. Preparation for TEM re-treatment was carried out by incubating the PC3^res^ cells for three days with TEM-free medium. Subsequently, 10 nm of TEM was applied to the PC3^res^ and PC3^par^ cells, and cell cultures were then subjected to the below assays.

### 2.3. Tumor Cell Binding to HUVECs

To analyze tumor cell adhesion, HUVECs were transferred to six-well multiplates (Falcon Primaria; BD Biosciences, Franklin Lakes, NJ, USA) in complete HUVEC medium. When confluency was reached, PC3^par^ or PC3^res^ cells were detached from the culture flasks by accutase treatment (PAA Laboratories, Cölbe, Germany) and 0.5 × 10^6^ cells were then added to the HUVEC monolayer for 30, 60, or 120 min. Subsequently, non-adherent tumor cells were washed off using warmed (37 °C) Medium 199. The remaining cells were fixed with 1% glutaraldehyde. Adherent tumor cells (appearing as rounded, light cells) were counted in five different fields (5 × 0.25 mm^2^) using a phase contrast microscope and the mean cellular adhesion rate was calculated.

### 2.4. Attachment to Extracellular Matrix Components

Six-well plates were coated with collagen G (extracted from calfskin, consisting of 90% collagen type I and 10% collagen type III (Seromed) diluted to 400 µg/mL in phosphate buffered saline (PBS)), laminin (derived from the Engelbreth–Holm–Swarm mouse tumor, diluted to 50 µg/mL in PBS; BD Biosciences), or fibronectin (derived from human plasma; diluted to 50 µg/mL in PBS; BD Biosciences) overnight. Unspecific cell binding was evaluated by culture plates treated with poly-d-lysine (Nunc, Wiesbaden, Germany). Plastic dishes served as the background control. Plates were washed with 1% bovine serum albumin (BSA) in PBS to block nonspecific cell adhesion. Then, 0.5 × 10^6^ tumor cells were added to each well for 60 min. Subsequently, non-adherent cells were fixed with 1% glutaraldehyde and counted microscopically. The mean cellular adhesion rate, defined by the number of cells which adhered to the coated wells minus the number of cells which adhered to the non-coated wells (background), was calculated from five different observation fields (5 × 0.25 mm^2^).

### 2.5. Tumor Cell Motility (Chemotaxis), Migration, and Invasion

Serum-induced chemotactic movement was examined using six-well Transwell chambers (Greiner, Frickenhausen. Germany) with 8-µm pores. Either 0.5 × 10^6^ PC3^par^ or PC3^res^ cells per mL were placed in an upper chamber in serum-free medium. To evaluate cell migration, Transwell chambers were pre-coated with collagen (400 µg/mL). Cell invasion was investigated by coating the Transwell chambers with collagen (400 µg/mL), which were then overlaid with HUVECs. The lower chamber contained 10% serum. After 20 h of incubation, the upper surface of the Transwell membrane was gently wiped with a cotton swab to remove non-migrating cells. Cells that had moved to the lower surface of the membrane were stained using hematoxylin and counted microscopically. The mean chemotaxis, migration, or invasion rate was calculated from five different observation fields (5 × 0.25 mm^2^).

### 2.6. Integrin Surface Expression

Integrin surface expression was compared on PC3^par^ and PC3^res^ cells, and on PC3^par^ and PC3^res^ cells treated with VPA. Tumor cells were washed in blocking solution (PBS, 0.5% BSA) and then incubated for 60 min at 4 °C with phycoerythrin (PE)-conjugated monoclonal antibodies directed against the following integrin subtypes: anti-α1 (IgG1; clone SR84, dilution 1:1000), anti-α2 (IgG2a; clone 12F1-H6, dilution 1:250), anti-α3 (IgG1; clone C3II.1, dilution 1:1000), anti-α4 (IgG1; clone 9F10, dilution 1:200), anti-α5 (IgG1; clone IIA1, dilution 1:5000), anti-α 6 (IgG2a; clone GoH3, dilution 1:200), anti-β1 (IgG1; clone MAR4, dilution 1:2500), anti-β3 (IgG1; clone VI-PL2, dilution 1:2500), or anti-β4 (IgG2a; clone 439-9B, dilution 1:250; all BD Biosciences). Integrin surface expression was then measured using FACscan (BD Biosciences; FL-2H (log) channel histogram analysis; 1 × 10^4^ cells per scan) and expressed as mean fluorescence units (MFU). A mouse IgG1-PE (MOPC-21) or IgG2a-PE (G155-178; all: BD Biosciences) was used as an isotype control.

### 2.7. Western Blot Analysis

To explore the integrin proteins in PC3^par^ and PC3^res^ cells, tumor cell lysates were applied to a 7% polyacrylamide gel and electrophoresed for 90 min at 100 V. The protein was then transferred to nitrocellulose membranes (1 h, 100 V). After blocking with non-fat dry milk for 1 h, the membranes were incubated overnight with the monoclonal antibodies listed above (unconjugated). Additionally, integrin-related signaling was investigated using anti-integrin-linked kinase (ILK; clone 3, dilution 1:1000) and anti-phospho-specific focal adhesion kinase (FAK; pY397; clone 18, dilution 1:1000) antibodies (all: BD Biosciences).

To evaluate the target specificity of TEM and VPA, the following monoclonal antibodies were employed: Anti Akt, anti-phospho Akt (pAkt; clone 104A282, both: mouse IgG1, dilution 1:500, BD Biosciences), and anti-acetylated H3 (aH3; rabbit IgG, clone Y28, dilution 1:500, Epitomics, USA). Horseradish peroxidase (HRP) -conjugated goat-anti-mouse IgG (Upstate Biotechnology, Lake Placid, NY, USA; dilution 1:5000) served as the secondary antibody. Membranes were briefly incubated with enhanced chemiluminescence (ECL) detection reagent (ECLTM, Amersham/GE Healthcare, München, Germany) to visualize the proteins and then analyzed by the Fusion FX7 system (Peqlab, Erlangen, Germany). β-actin (1:1000; Sigma, Taufenkirchen, Germany) served as the internal control. Gimp 2.8 software was used to perform pixel density analysis of the protein bands and to calculate the ratio of protein intensity/β-actin intensity.

### 2.8. Real-Time (RT)-qPCR

RT-qPCR was done in triplicate. cDNA synthesis was performed using 3 µg of total RNA per sample according to the manufacturer’s protocol by AffinityScript QPCR cDNA Synthesis Kit (Stratagene, Amsterdam, the Netherlands). Quantitative gene expression analysis by RT-PCr was performed by the Mx3005p (Stratagene) using SYBER-Green Super Array (SABioscience Corporation, Valencia, CA, USA) and SuperArray primer sets: *GPDH* (NM_002046.3, Hs.592355), *integrin* α1 (ITGA1, NM_181501, Hs.644652), *integrin* α2 (ITGA2, NM_02203, Hs.482077), *integrin* α3 (ITGA3, NM_002204, Hs.265829), *integrin* α4 (ITGA4, NM_000885, Hs. 694732), *integrin* α5 (ITGA5, NM_002205, Hs. 505654), *integrin* α6 (ITGA6, NM_000210, Hs.133397), *integrin* β1 (ITGB1, NM_002211, Hs.643813), *integrin* β3 (ITGB3, NM_000212, HS218040), and *integrin* β4 (ITGB4, NM_000213, Hs.632226; all SABioscience Corporation). Calculation of the relative expression of each gene was done by the ΔΔCt method in the analysis program from SABioscience Corporation. The housekeeping gene, *GAPDH*, was used for normalization.

### 2.9. Blocking Studies

To determine whether integrin α2, α5, and β1 impact metastatic spread, drug-sensitive or -resistant cells were incubated for 60 min with 10 µg/mL function-blocking anti-integrin α2 (clone P1E6) mouse mAb, anti-integrin α5 (clone P1D6) mouse mAb, or anti-integrin β1 (clone 6S6) mouse mAB (all: from Millipore, Burlington, MA, USA), respectively. Controls were incubated with cell culture medium alone. Subsequently, tumor cell adhesion to immobilized collagen, as well as chemotaxis and migration, were evaluated as described above.

### 2.10. Statistics

All experiments were performed 3–6 times. Statistical significance was determined with the Wilcoxon–Mann-Whitney-U-test. Differences were considered statistically significant at a *p*-value less than 0.05.

## 3. Results

### 3.1. Adhesion Characteristics

**Figure 1:**
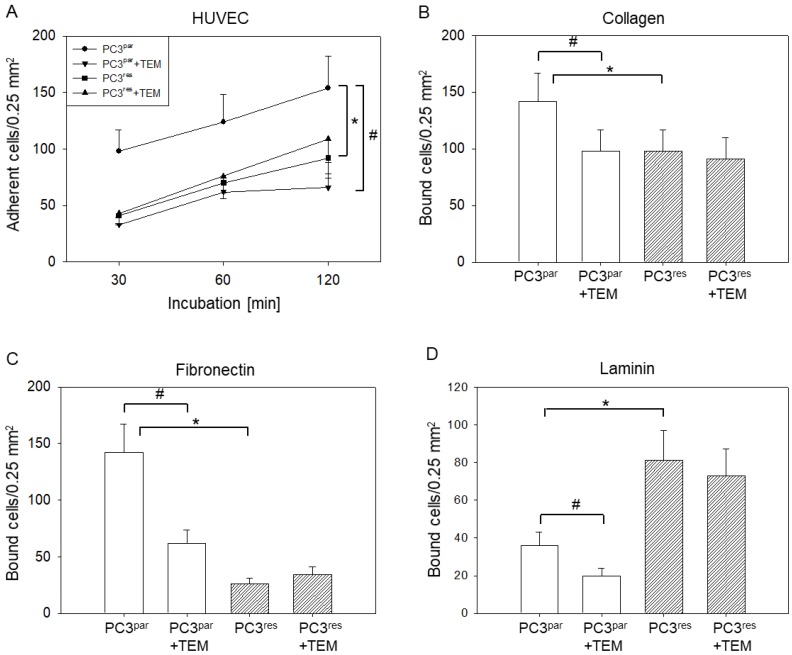
Adhesion of temsirolimus (TEM)-resistant (PC3^res^) and TEM-sensitive (PC3^par^) prostate cancer cells. (**A**) time-dependent PC3 adhesion to human vein endothelial cells (HUVEC); (**B**) binding to immobilized collagen; (**C**) binding to immobilized fibronectin; (**D**) binding to immobilized laminin. * indicates significant difference to the temsirolimus-free control (PC3^par^). # indicates significant difference between cells exposed to 10 nm of TEM and untreated cells.

More PC3^par^ cells adhered to HUVECs over time than did PC3^res^ cells (Figure 1A). The application of 10 nm of TEM resulted in a diminished attachment rate of PC3^par^ but not of PC3^res^ cells. Tumor cell interaction with an extracellular matrix revealed lower adhesion of PC3^res^ cells to immobilized collagen (Figure 1B) or fibronectin (Figure 1C), but an increased adhesion to laminin (Figure 1D), compared to the parental cells. Treating the tumor cells with TEM (10 nm) suppressed binding of the drug-sensitive (PC3^par^) but not the drug-resistant (PC3^res^) cells to collagen, fibronectin, and laminin.

### 3.2. Tumor Motility, Migration, and Invasion

**Figure 2:**
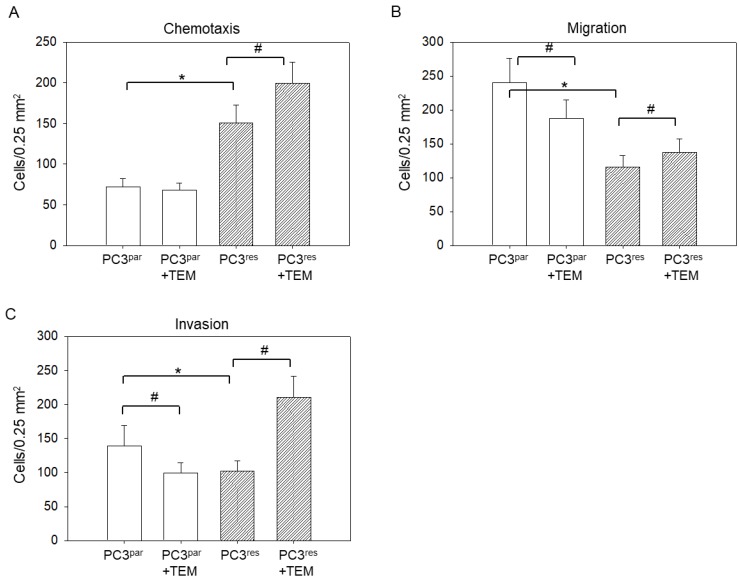
Motility of temsirolimus (TEM)-resistant (PC3^res^) and TEM-sensitive (PC3^par^) prostate cancer cells. Mean values were calculated from five counts. (**A**) PC3 chemotaxis; (**B**) PC3 migration; (**C**) PC3 invasion. * indicates significant difference to the temsirolimus-free control (PC3^par^). # indicates significant difference between the treated and untreated PC3 sublines with 10 nm TEM.

Chemotaxis was significantly elevated in PC3^res^ versus PC3^par^ cells (Figure 2A), whereas motile spreading through a collagen matrix (migration, Figure 2B) or through HUVECs layered onto collagen (invasion, Figure 2C) was diminished. TEM blocked migration and invasion of PC3^par^ but not of PC3^res^ cells. Rather, chemotaxis, migration, and invasion of PC3^res^ cells were even enhanced following TEM re-treatment.

### 3.3. Integrin Expression Pattern in PC3^par^ and PC3^res^ Cells

**Figure 3:**
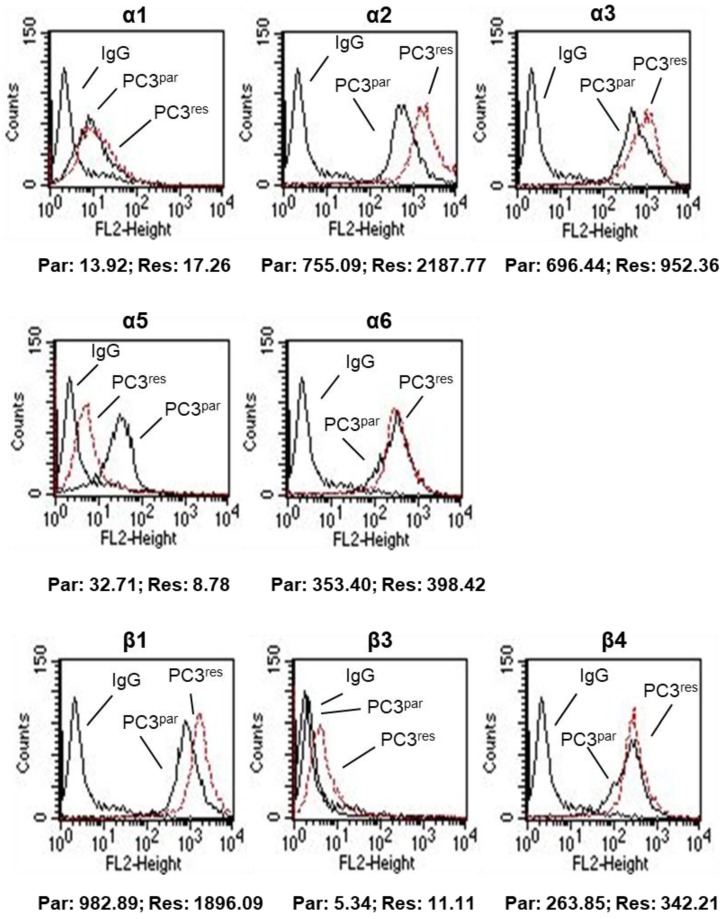
Flow activated cell sorting (FACS) analysis of integrin **α** and **β** subtype expression on temsirolimus (TEM)-resistant (PC3^res^) and TEM-sensitive (PC3^par^) prostate cancer cells. Mean fluorescence values are shown below the histograms (Par = PC3 parental cells, Res = PC3-resistant cells). One of three independent experiments.

The integrin subtypes α2, α3, α6, β1, and β4 were strongly expressed, α1 and α5 were moderately expressed, and β3 was expressed to a low extent in PC3^par^ cells (Figure 3). The α4 integrin subtype was not detectable by flow cytometry, neither on PC3^par^ nor on PC3^res^ cells (data not shown). PC3^res^ cells were characterized by distinct differences in the integrin-expression pattern, compared to controls. The α2, α3, β1, and β4 subtypes were distinctly elevated, whereas α5 was nearly lost on the PC3^res^ cell membrane. β3 was also found to be (moderately) up-regulated in the resistant cell population. No significant differences in expression were seen with respect to integrin α1 or α6 (PC3^par^ versus PC3^res^).

**Figure 4:**
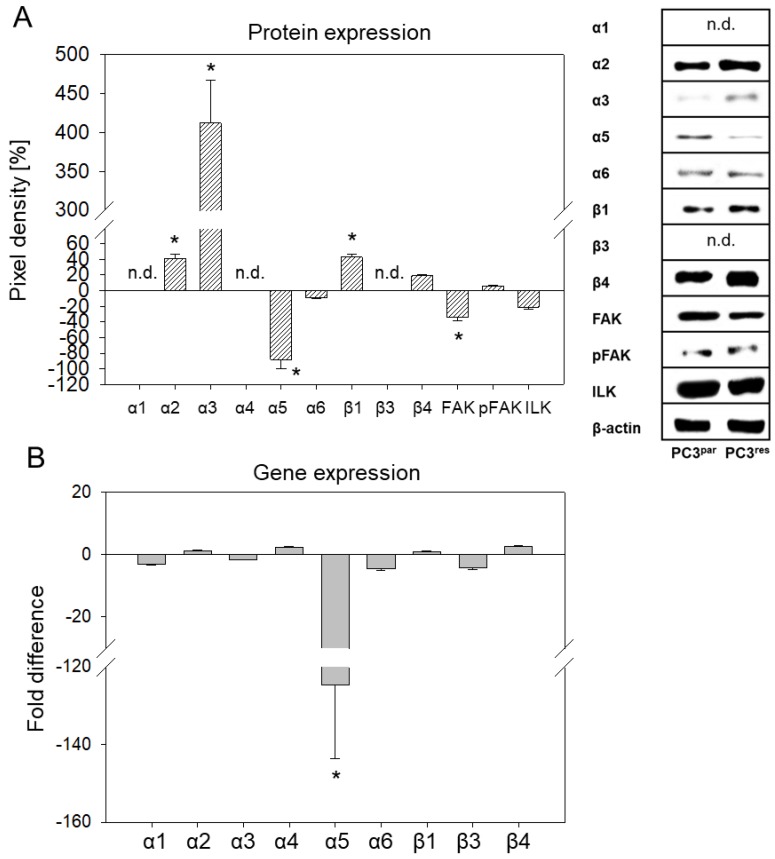
(**A**) Integrin protein level in temsirolimus-resistant (PC3^res^) versus temsirolimus-sensitive (PC3^par^) cells quantified by pixel density analysis. Integrins were evaluated three times, integrin-linked kinase (ILK), focal adhesion kinase (FAK), and phosphorylated focal adhesion kinase (pFAK) four times. Representative Western blots are shown on the right panel. (**B**) The integrin gene expression pattern in PC3^res^ versus PC3^par^ cells. Values are given as fold difference to PC3^par^ cells. * indicates a significant difference.

Western blotting demonstrated enhanced protein content of integrins α2, α3, β1, and β4 in PC3^res^ cells, as compared to the control cell line (PC3^par^). The α5 integrin was considerably suppressed in PC3^res^ cells. No differences were seen with integrin α6. The integrin members α1 and β3 were not detectable. FAK was slightly diminished, whereas pFAK and ILK were equally expressed in PC3^par^ and PC3^res^ cells (Figure 4A).

α5 *integrin* mRNA was expressed in PC3^res^ at a very low level compared to the PC3^par^ cells (Figure 4B). The mRNA of the other integrin subtypes displayed no significant differences between the sensitive and resistant cells.

### 3.4. Blocking Studies

Blocking studies were carried out to investigate the function of α2 and β1 integrins, which were strongly elevated in PC3^res^ compared to PC3^par^, and to explore the mode of action of integrin α5, which was distinctly diminished in the resistant cell population.

**Figure 5:**
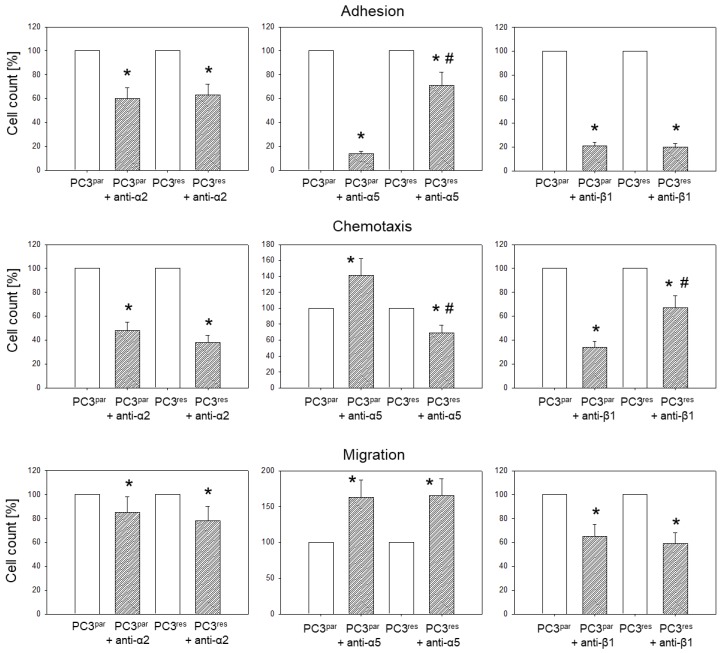
Influence of integrin α2, α5, or β1 blockade on PC3 adhesion, chemotaxis, and migration. Values are shown as percentage difference to their respective 100% controls. * indicates significant difference between the PC3 control subline and the PC3 subline treated with the function-blocking antibody. # indicates significant difference between temsirolimus-sensitive (PC3^par^) and temsirolimus-resistant (PC3^res^) cells whose integrin subtype was blocked.

Blocking α2 or β1 significantly down-regulated adhesion, chemotactic movement, and migration of both PC3^res^ and PC3^par^ cells. The effect of receptor blockade on both cell sublines was similar, excepting chemotaxis, where β1 influenced PC3^par^ cells more efficiently than PC3^res^ cells (Figure 5). Blockade of integrin α5 differentially altered cell behavior. Adhesion of PC3^par^ to collagen was drastically reduced, while adhesion of PC3^res^ was only moderately diminished. Migration of PC3^res^ and PC3^par^ increased to a similar extent. However, chemotaxis of PC3^par^ was up-regulated, whereas activity of PC3^res^ was down-regulated.

### 3.5. Influence of VPA on Adhesion, Chemotaxis, Migration, and Integrin Expression of PC3^par^ and PC3^res^ Cells

**Figure 6:**
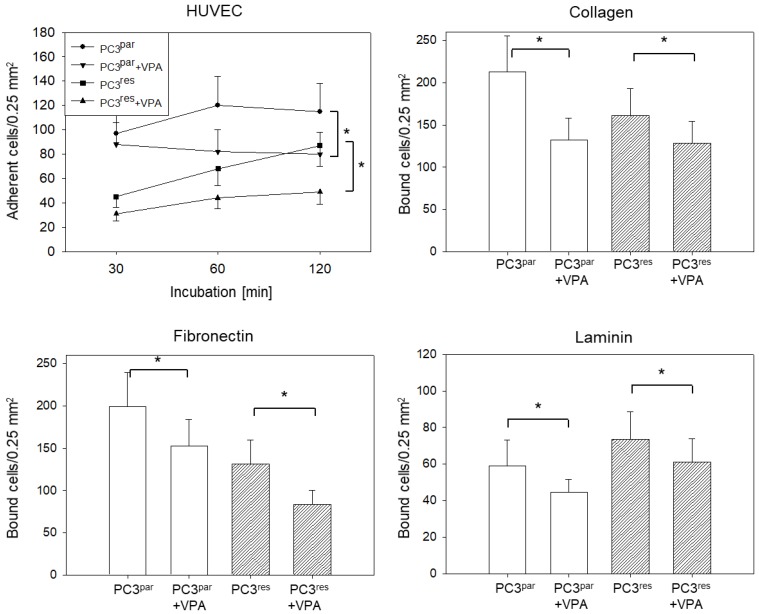
Adhesion of temsirolimus (TEM)-resistant (PC3^res^) versus TEM-sensitive (PC3^par^) prostate cancer cells in the presence of valproic acid (VPA). The figure depicts time-dependent PC3 adhesion to human umbilical vein endothelial cells (HUVEC), binding to immobilized collagen, fibronectin, or laminin. * indicates significant difference to controls not treated with VPA.

**Figure 7:**
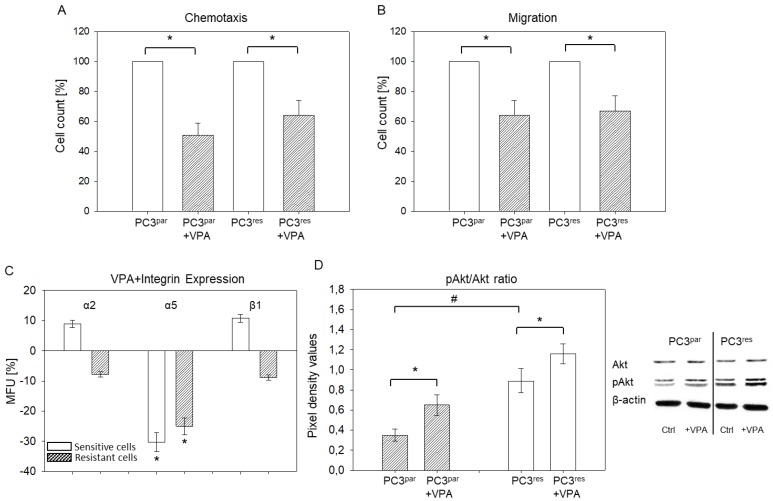
(**A**,**B**). Chemotactic movement and migration of PC3^res^ versus PC3^par^ cells treated with valproic acid (VPA). Values are given as percentage difference to their respective 100% controls. * indicates significant difference to controls not treated with VPA. (**C**). Influence of VPA on integrin α2, α5, or β1 expression. Mean fluorescence units (MFU) are shown as percentage difference to the respective 100% controls (not treated with VPA). (**D**) Influence of VPA on Akt expression. Akt and pAkt levels were quantified by Western blotting and pixel density analysis. Pixel density values of the pAkt/Akt ratio and representative Western blots are shown (Ctrl = control). * indicates significant difference between the PC3 control subline and the PC3 subline treated with VPA. # indicates significant difference between PC3^par^ and PC3^res^ cells.

VPA significantly down-regulated tumor cell binding to immobilized collagen, fibronectin, or matrigel of both PC3^par^ and PC3^res^ cells, as compared to the untreated controls (Figure 6). The same was true with respect to tumor cell attachment to HUVECs. Chemotactic movement and migration were also diminished when VPA was applied to drug-sensitive or drug-resistant tumor cells (Figure 7A,B). Integrin expression in the presence of VPA revealed a significant down-regulation of α5 in both PC3^par^ and PC3^res^ cells. Figure 7C depicts percentage difference of integrin expression level in VPA-treated cells, compared to the controls set to 100%. Figure 7D shows that VPA also acts on pAkt expression in both PC3^par^ and PC3^res^ cells. VPA did not induce toxic effects, as has been demonstrated by the trypan dye exclusion test (data not shown). Since VPA serves as an HDAC inhibitor, this was proved by staining VPA-treated PC3 cells with an anti-acetylated histone H3 (aH3) antibody. Pixel density analysis demonstrated an increase of aH3 to 205% (PC3^par^) and 199% (PC3^res^), as compared to PC3^par^ and PC3^res^ cells not treated with VPA (set to 100%).

## 4. Discussion

Prostate cancer cell adhesion, chemotaxis, and invasion were altered after TEM resistance developed. Tumor–HUVEC and tumor–matrix interactions were significantly blocked by TEM application to the drug-sensitive cells but not to the resistant PC3 cells. Binding of PC^res^ to immobilized laminin was considerably elevated compared to PC^par^ binding, corroborating studies from Liberio et al. where an increased laminin attachment of prostate cancer cells was associated with increased cell mobility [18]. Conversely, adhesion of PC3^res^ to endothelium, as well as to collagen and fibronectin, was significantly decreased, as compared to PC3^par^ cells. Earlier studies have shown that a loss of fibronectin binding promotes invasion by facilitating the detachment of cancer cells from the tumor mass [19]. Apparently, TEM resistance diminishes the (firm) contact of prostate cancer cells to the vascular wall and the underlying matrix proteins, collagen and fibronectin, while strengthening the laminin contact. These mechanisms serve as a prerequisite to metastatic progression.

The cell migration (through collagen) and invasion assay (through collagen and HUVECs) carried out in the present investigation could contradict the hypothesis that TEM-induced-resistance enhances motile crawling, since the number of counted PC3^res^ cells was lower than that for PC3^par^ cells. This could to be due to inhibited cell adhesion of PC3^res^ cells to collagen or HUVECs, compared to PC3^par^ cells, and consequently lead to a lower number of migrating cells. In fact, treating PC3^res^ cells with TEM significantly increased their migration and invasion potential, and chemotaxis strongly increased during resistance development, particularly when PC3 were re-treated with therapeutically relevant TEM concentrations, further activating their invasive behavior.

TEM resistance, therefore, may not only be coupled to “non-responsiveness” but rather to re-activation of the cellular motor machinery, leading to a highly invasive tumor phenotype. Chronic treatment of prostate cancer cells with another mTOR inhibitor, everolimus, has also been associated with increased metastatic activity [20]. The same increase in metastatic activity has been observed in renal cell carcinoma cells with acquired resistance towards TEM [21]. It is not yet clear whether mTOR represents the pivotal element triggering invasion. However, Caino and Altieri recently pointed to the close correlation between mTOR signaling and the crawling velocity of tumor cells [22]. Neuroblastoma and breast carcinoma models have provided evidence that mTOR activates pathways relevant to motility regulation [23,24].

Accelerated invasion was accompanied by a change in the integrin expression pattern. Both cell surface and cytoplasmic protein levels of α2- and β1- integrin increased, whereas α5 decreased in PC3^res^ versus PC3^par^ cells. Analysis of the integrin coding genes demonstrated a significant down-regulation of α5, indicating that this integrin subtype has been modified on a transcriptional level.

The role of α2 in prostate cancer is controversial. Current and former functional blocking studies have demonstrated a positive association between α2 expression and adhesion and migration properties [20,25]. In contrast, Ramirez et al. postulated that α2 integrin may act as a tumor suppressor and loss of it may induce bone metastasis [26]. However, this postulate was exclusively related to the expression of the α2 encoding gene. Since no change in the α2 gene was observed during the present investigation, an epigenetic mechanism behind α2 up-regulation under chronic drug treatment may be assumed. It is concluded that elevated α2 may promote cancer metastatic potential through activation of integrin-mediated signaling pathways. Accordingly, Van Slambrouck et al. showed that a high α2 expression level is necessary to maintain a highly invasive phenotype of prostate cancer cells [27]. A clinical investigation has presented evidence of elevated α2 in skeletal metastases, compared to primary prostate cancer lesions [28].

The integrin subtype β1 was similarly modified to α2 during resistance development. This may be expected, since β1 interacts with α2 to form a functional unit [28,29]. Still, the effect of β1 is controversial. Experiments on PC3 prostate cancer cells have shown that β1-integrin may stimulate tumor growth or invasion by dynamically regulating inside-out-signaling, while reduced β1-signaling may promote metastatic dissemination [30]. On the other hand, knockdown of β1 in the same cell line (PC3) significantly inhibits tumor cell migration [31], and overexpression is particularly apparent in human bone metastases [32], underpinning β1’s role to drive invasive processes. Interestingly, the β1-integrin has recently been reported to control skeletal metastasis by crosstalking with the Akt–mTOR pathway [33,34], which might explain why chronic TEM application is coupled to both increased tumor invasiveness and increased β1-integrin expression. The Akt–mTOR pathway was not investigated in the present study. However, chronic TEM exposure has previously been shown to re-activate Akt-mTOR signaling [17]. It might, therefore, be likely that integrin β1 becomes up-regulated due to direct communication with the Akt–mTOR pathway, allowing tumor cells to begin the invasive cascade.

Tesfay et al. have concluded from their studies that autophagy loss may be coupled to intracellular β1-integrin accumulation caused by lacking lysosomal integrin degradation [35]. Dower and coworkers assumed that integrin-mediated regulation of cell migration and invasion might depend on endosome-mediated trafficking and integrin degradation [36]. Therefore, the higher protein levels of β1, along with α2, α3, and β4, in PC3^res^ cells might be due to disrupted autophagy induced by chronic mTOR inhibition. Still this is hypothetical and requires further investigation. In fact, an integrin β1 protein increase combined with an increased α3 protein level has been considered to activate prostate cancer cell migration and invasion by regulating downstream signaling [37], whereby α3 might be a responsible triggering factor in controlling cell motility [38].

Integrin α5 was not only down-regulated in PC3^res^ cells but was also found to be reduced in TEM-resistant renal cell carcinoma cells [21] or in prostate cancer cells with acquired resistance to everolimus [20]. This might reflect a generalized response to chronic mTOR-blockade, although the relevance of this behavior is not totally clear. Examination of 157 prostate cancer cases has revealed a negative correlation between α5 expression and Gleason score, pathological stage, lymph node metastasis, and prostate-specific antigen level [39], indicating that loss of α5 is associated with tumor progression. Based on an earlier study, it was hypothesized that resistance causes a functional switch of α5, driving the tumor cells from adhesiveness to invasiveness [20]. In fact, the present investigation demonstrates that α5 strongly regulates adhesion of TEM-sensitive tumor cells, while playing a major role in controlling migration during drug resistance. Since integrin α5 serves as a fibronectin receptor, this can explain why reduced α5 expression on PC3^res^ was associated with cell detachment from the fibronectin matrix.

Attention should also be paid to the elevated surface expression of the laminin receptor integrin α3 in PC3^res^ (versus PC3^par^) cells, which correlated with enhanced tumor binding to laminin. Although the relevance of α3 on metastatic progression of prostate cancer has not been fully elucidated, in vitro experiments have recently demonstrated that α3 may promote survival of laminin-adherent tumor cells [35] and loss of α3 may impair adhesion to laminin [40]. Resistance development to the mTOR inhibitor TEM might, therefore, be accompanied by both a functional and quantitative modulation of the integrin subtype α5, associated with an α3 elevation, leading to an adhesive switch from fibronectin to laminin.

HDAC inhibition significantly blocked tumor cell adhesion and invasion of both TEM-sensitive and TEM-resistant PC3 cells. This finding is important, since it indicates that chemoresistant tumor patients may profit from HDAC targeting. We did not investigate histone acetylation in this study but have demonstrated earlier that VPA acetylates histones H3 and H4 in TEM-resistant bladder cancer [17] and renal cancer cells driven to everolimus non-responsiveness [9], indicating its role as an epigenetic modulator. Indeed, VPA has been reported to revert epithelial-mesenchymal-transition of mTOR inhibitor resistant tumor cells by targeting histone deacetylases [41].

VPA induced a strong integrin α5 decrease in both PC3^res^ and PC3^par^ cells, which was associated with an equally strong decrease in chemotaxis. The loss of α5 induced by TEM, on the other hand, was associated with increased chemotaxis. To account for the opposing chemotaxic effect the two drugs exert, it is important to remember that different signaling pathways are modified by TEM and VPA. Pan et al. recently pointed out that regulation of tumor cell behavior by integrins depends on how the receptors affect cell signaling [42]. TEM resistance has been shown to be coupled to a strong phosphorylation of Akt and the mTOR complex Raptor, along with c-Met activation [17,43,44]. This is relevant, since c-Met has recently been demonstrated to displace α5 integrin from β1 integrin during resistance development. This α5 loss is associated with increased invasion [45].

VPA has been shown to act on histone acetylation as its primary target, thereby reverting epithelial–mesenchymal transition and blocking tumor cell migration, both associated with α5 reduction [46,47]. Therefore, it may be assumed that invasion blocking by VPA, used here as the prototype HDAC inhibitor, is at least partially caused by suppression of α5 integrin expression (although further experiments are required for confirmation). The clinical importance of this has been demonstrated by Siva et al. who have recently identified α5 as a clinically relevant target for treating prostate cancer [48]. A peptide targeting activated α5β1 integrin has been developed, inducing complete regression of intramuscular prostate tumors in mice [49]. VPA use (in disorders for which a VPA indication exists) has been associated with a decreased cancer risk [50,51], and targeting HDAC is thought to be beneficial in treating prostate cancer, particularly in its advanced state [52,53]. In good accordance with our data, HDAC inhibition suppressed epithelial–mesenchymal plasticity and stemness activity observed in mesenchymal-like tumor cells resistant to Akt-mTOR pathway inhibitors [54]. This would support the role of HDAC inhibitors as a supplement to an mTORinhibitor based regimen.

VPA unexpectedly caused up-regulation of pAkt in both PC3^res^ and PC3^par^ cells. VPA has already been proven to block tumor cell growth and proliferation, even in chemo-resistant cells [17,55]. Therefore, elevation of pAkt by VPA may not be associated with increased growth activity of PC3 cells. Shi et al. presented evidence of reduced pAkt expression in PC3 cells caused by the HDAC inhibitor suberoylanilide hydroxamic acid (SAHA) [56]. In the same cell line, the HDAC inhibitor MS-275 (Entinostat) exerted only a marginal effect [57], and applying panobinostat (LBH589) did not lead to altered pAkt expression in PC3 cells [58]. Ellis et al. has shown that treating PC3 cells with panobinostat for 24 h down-regulated pAkt, whereas it was up-regulated with 48 h treatment [59]. This is of interest since VPA was also applied long-term in the present study (72 h) and likewise showed up-regulation. pAkt expression may, therefore, depend on the drug exposure time. Yan et al. has observed reduced pAkt expression in the presence of SAHA or panobinostat [60]. However, their results stem from the use of C4-2 cells, which are androgen receptor-positive, whereas PC3 cells are not. The communication between HDAC and Akt therefore seems complex and may depend on the tumor cell line, the androgen receptor status, and the applied HDAC inhibitor. Ongoing studies have been planned to deal with the Akt–mTOR cascade in more detail, to explore whether Akt up-regulation due to VPA exposure reflects a negative feedback loop or an unspecific epi-phenomenon.

Based on an in vitro model, evidence is presented here indicating that epigenetic suppression of the integrin α5 subtype could provide a novel strategy to combat progressive prostate cancer. Verification through animal studies would be the next step with regard to use of the HDAC-mTOR inhibitor combination. Although this combination has already been demonstrated to delay resistance in renal cell cancer cells, the protocol must be tailored to the prostate model. Finally, integrin α5 expression must be evaluated in tissue specimens derived from tumor patients.

## References

[1] Siegel R.L., Miller K.D., Jemal A. (2018). Cancer statistics, 2018. CA Cancer J. Clin..

[2] Ritch C.R., Cookson M.S. (2016). Advances in the management of castration resistant prostate cancer. BMJ.

[3] Calimeri T., Ferreri A.J.M. (2017). m-TOR inhibitors and their potential role in haematological malignancies. Br. J. Haematol..

[4] Pinto-Leite R., Arantes-Rodrigues R., Sousa N., Oliveira P.A., Santos L. (2016). mTOR inhibitors in urinary bladder cancer. Tumour Biol..

[5] Ciccarese C., Massari F., Iacovelli R., Fiorentino M., Montironi R., Di Nunno V., Giunchi F., Brunelli M., Tortora G. (2017). Prostate cancer heterogeneity: Discovering novel molecular targets for therapy. Cancer Treat. Rev..

[6] Yan G., Ru Y., Wu K., Yan F., Wang Q., Wang J., Pan T., Zhang M., Han H., Li X. (2018). GOLM1 promotes prostate cancer progression through activating PI3K-AKT-mTOR signaling. Prostate.

[7] Statz C.M., Patterson S.E., Mockus S.M. (2017). mTOR Inhibitors in Castration-Resistant Prostate Cancer: A Systematic Review. Target Oncol..

[8] Tsaur I., Makarević J., Hudak L., Juengel E., Kurosch M., Wiesner C., Bartsch G., Harder S., Haferkamp A., Blaheta R.A. (2011). The cdk1-cyclin B complex is involved in everolimus triggered resistance in the PC3 prostate cancer cell line. Cancer Lett..

[9] Juengel E., Nowaz S., Makarevi J., Natsheh I., Werner I., Nelson K., Reiter M., Tsaur I., Mani J., Harder S. (2014). HDAC-inhibition counteracts everolimus resistance in renal cell carcinoma in vitro by diminishing cdk2 and cyclin A. Mol. Cancer.

[10] Armstrong A.J., Shen T., Halabi S., Kemeny G., Bitting R.L., Kartcheske P., Embree E., Morris K., Winters C., Jaffe T. (2013). A phase II trial of temsirolimus in men with castration-resistant metastatic prostate cancer. Clin. Genitourin. Cancer.

[11] Qian C., Lai C.J., Bao R., Wang D.G., Wang J., Xu G.X., Atoyan R., Qu H., Yin L., Samson M. (2012). Cancer network disruption by a single molecule inhibitor targeting both histone deacetylase activity and phosphatidylinositol 3-kinase signaling. Clin. Cancer Res..

[12] Beagle B.R., Nguyen D.M., Mallya S., Tang S.S., Lu M., Zeng Z., Konopleva M., Vo T.T., Fruman D.A. (2015). mTOR kinase inhibitors synergize with histone deacetylase inhibitors to kill B-cell acute lymphoblastic leukemia cells. Oncotarget.

[13] Thelen P., Krahn L., Bremmer F., Strauss A., Brehm R., Loertzer H. (2013). Synergistic effects of histone deacetylase inhibitor in combination with mTOR inhibitor in the treatment of prostate carcinoma. Int. J. Mol. Med..

[14] Zibelman M., Wong Y.N., Devarajan K., Malizzia L., Corrigan A., Olszanski A.J., Denlinger C.S., Roethke S.K., Tetzlaff C.H., Plimack E.R. (2015). Phase I study of the mTOR inhibitor ridaforolimus and the HDAC inhibitor vorinostat in advanced renal cell carcinoma and other solid tumors. Investig. New Drugs.

[15] Dembla V., Groisberg R., Hess K., Fu S., Wheler J., Hong D.S., Janku F., Zinner R., Piha-Paul S.A., Ravi V. (2017). Outcomes of patients with sarcoma enrolled in clinical trials of pazopanib combined with histone deacetylase, mTOR, Her2, or MEK inhibitors. Sci. Rep..

[16] Booth L., Roberts J.L., Sander C., Lee J., Kirkwood J.M., Poklepovic A., Dent P. (2017). The HDAC inhibitor AR42 interacts with pazopanib to kill trametinib/dabrafenib-resistant melanoma cells in vitro and in vivo. Oncotarget.

[17] Juengel E., Najafi R., Rutz J., Maxeiner S., Makarevic J., Roos F., Tsaur I., Haferkamp A., Blaheta R.A. (2017). HDAC inhibition as a treatment concept to combat temsirolimus-resistant bladder cancer cells. Oncotarget.

[18] Liberio M.S., Sadowski M.C., Soekmadji C., Davis R.A., Nelson C.C. (2014). Differential effects of tissue culture coating substrates on prostate cancer cell adherence, morphology and behavior. PLoS ONE.

[19] Jia D., Entersz I., Butler C., Foty R.A. (2012). Fibronectin matrix-mediated cohesion suppresses invasion of prostate cancer cells. BMC Cancer.

[20] Tsaur I., Makarević J., Juengel E., Gasser M., Waaga-Gasser A.M., Kurosch M., Reiter M., Wedel S., Bartsch G., Haferkamp A. (2012). Resistance to the mTOR-inhibitor RAD001 elevates integrin α2- and β1-triggered motility, migration and invasion of prostate cancer cells. Br. J. Cancer.

[21] Juengel E., Makarević J., Reiter M., Mani J., Tsaur I., Bartsch G., Haferkamp A., Blaheta R.A. (2014). Resistance to the mTOR inhibitor temsirolimus alters adhesion and migration behavior of renal cell carcinoma cells through an integrin α5- and integrin β3-dependent mechanism. Neoplasia.

[22] Caino M.C., Altieri D.C. (2015). Cancer cells exploit adaptive mitochondrial dynamics to increase tumor cell invasion. Cell Cycle.

[23] Joly M.M., Williams M.M., Hicks D.J., Jones B., Sanchez V., Young C.D., Sarbassov D.D., Muller W.J., Brantley-Sieders D., Cook R.S. (2017). Two distinct mTORC2-dependent pathways converge on Rac1 to drive breast cancer metastasis. Breast Cancer Res..

[24] Hua Z., Gu X., Dong Y., Tan F., Liu Z., Thiele C.J., Li Z. (2016). PI3K and MAPK pathways mediate the BDNF/TrkB-increased metastasis in neuroblastoma. Tumour Biol..

[25] Van Slambrouck S., Jenkins A.R., Romero A.E., Steelant W.F. (2009). Reorganization of the integrin α2 subunit controls cell adhesion and cancer cell invasion in prostate cancer. Int. J. Oncol..

[26] Ramirez N.E., Zhang Z., Madamanchi A., Boyd K.L., O’Rear L.D., Nashabi A., Li Z., Dupont W.D., Zijlstra A., Zutter M.M. (2011). The α2β1 integrin is a metastasis suppressor in mouse models and human cancer. J. Clin. Investig..

[27] Van Slambrouck S., Hilkens J., Bisoffi M., Steelant W.F. (2009). AsialoGM1 and integrin α2β1 mediate prostate cancer progression. Int. J. Oncol..

[28] Sottnik J.L., Daignault-Newton S., Zhang X., Morrissey C., Hussain M.H., Keller E.T., Hall C.L. (2013). Integrin α2β1 (α2β1) promotes prostate cancer skeletal metastasis. Clin. Exp. Metastasis.

[29] Lee S.H., Hatakeyama S., Yu S.Y., Bao X., Ohyama C., Khoo K.H., Fukuda M.N., Fukuda M. (2009). Core3 *O*-glycan synthase suppresses tumor formation and metastasis of prostate carcinoma PC3 and LNCaP cells through down-regulation of α2β1 integrin complex. J. Biol. Chem..

[30] Pollan S.G., Huang F., Sperger J.M., Lang J.M., Morrissey C., Cress A.E., Chu C.Y., Bhowmick N.A., You S., Freeman M.R. (2018). Regulation of inside-out β1-integrin activation by CDCP1. Oncogene.

[31] Kurozumi A., Goto Y., Matsushita R., Fukumoto I., Kato M., Nishikawa R., Sakamoto S., Enokida H., Nakagawa M., Ichikawa T. (2016). Tumor-suppressive microRNA-223 inhibits cancer cell migration and invasion by targeting ITGA3/ITGB1 signaling in prostate cancer. Cancer Sci..

[32] Jin J.K., Tien P.C., Cheng C.J., Song J.H., Huang C., Lin S.H., Gallick G.E. (2015). Talin1 phosphorylation activates β1 integrins: A novel mechanism to promote prostate cancer bone metastasis. Oncogene.

[33] Yu M., Wang J., Muller D.J., Helenius J. (2015). In PC3 prostate cancer cells ephrin receptors crosstalk to β1-integrins to strengthen adhesion to collagen type I. Sci. Rep..

[34] Virtakoivu R., Pellinen T., Rantala J.K., Perälä M., Ivaska J. (2012). Distinct roles of AKT isoforms in regulating β1-integrin activity, migration, and invasion in prostate cancer. Mol. Biol. Cell.

[35] Tesfay L., Schulz V.V., Frank S.B., Lamb L.E., Miranti C.K. (2016). Receptor tyrosine kinase Met promotes cell survival via kinase-independent maintenance of integrin α3β1. Mol. Biol. Cell.

[36] Dower C.M., Wills C.A., Frisch S.M., Wang H.G. (2018). Mechanisms and context underlying the role of autophagy in cancer metastasis. Autophagy.

[37] Kurozumi A., Goto Y., Matsushita R., Fukumoto I., Kato M., Nishikawa R., Sakamoto S., Enokida H., Nakagawa M., Ichikawa T. (2016). Tumor-suppressive microRNA-223 inhibits cancer cell migration and invasion by targeting ITGA3/ITGB1 signaling in prostate cancer. Cancer Sci..

[38] Das L., Anderson T.A., Gard J.M., Sroka I.C., Strautman S.R., Nagle R.B., Morrissey C., Knudsen B.S., Cress A.E. (2017). Characterization of Laminin Binding Integrin Internalization in Prostate Cancer Cells. J. Cell. Biochem..

[39] Drivalos A., Chrisofos M., Efstathiou E., Kapranou A., Kollaitis G., Koutlis G., Antoniou N., Karanastasis D., Dimopoulos M.A., Bamias A. (2016). Expression of α5-integrin, α7-integrin, Ε-cadherin, and *N*-cadherin in localized prostate cancer. Urol. Oncol..

[40] Varzavand A., Drake J.M., Svensson R.U., Herndon M.E., Zhou B., Henry M.D., Stipp C.S. (2013). Integrin α3β1 regulates tumor cell responses to stromal cells and can function to suppress prostate cancer metastatic colonization. Clin. Exp. Metastasis.

[41] Juengel E., Dauselt A., Makarević J., Wiesner C., Tsaur I., Bartsch G., Haferkamp A., Blaheta R.A. (2012). Acetylation of histone H3 prevents resistance development caused by chronic mTOR inhibition in renal cell carcinoma cells. Cancer Lett..

[42] Pan B., Guo J., Liao Q., Zhao Y. (2018). β1 and β3 integrins in breast, prostate and pancreatic cancer: A novel implication. Oncol. Lett..

[43] Imura Y., Yasui H., Outani H., Wakamatsu T., Hamada K., Nakai T., Yamada S., Myoui A., Araki N., Ueda T. (2014). Combined targeting of mTOR and c-MET signaling pathways for effective management of epithelioid sarcoma. Mol. Cancer.

[44] Etnyre D., Stone A.L., Fong J.T., Jacobs R.J., Uppada S.B., Botting G.M., Rajanna S., Moravec D.N., Shambannagari M.R., Crees Z. (2014). Targeting c-Met in melanoma: Mechanism of resistance and efficacy of novel combinatorial inhibitor therapy. Cancer Biol. Ther..

[45] Jahangiri A., Nguyen A., Chandra A., Sidorov M.K., Yagnik G., Rick J., Han S.W., Chen W., Flanigan P.M., Schneidman-Duhovny D. (2017). Cross-activating c-Met/β1 integrin complex drives metastasis and invasive resistance in cancer. Proc. Natl. Acad. Sci. USA.

[46] Makarević J., Tawanaie N., Juengel E., Reiter M., Mani J., Tsaur I., Bartsch G., Haferkamp A., Blaheta R.A. (2014). Cross-communication between histone H3 and H4 acetylation and Akt-mTOR signalling in prostate cancer cells. J. Cell. Mol. Med..

[47] Wedel S., Hudak L., Seibel J.M., Makarević J., Juengel E., Tsaur I., Wiesner C., Haferkamp A., Blaheta R.A. (2011). Impact of combined HDAC and mTOR inhibition on adhesion, migration and invasion of prostate cancer cells. Clin. Exp. Metastasis.

[48] Siva A.C., Kirkland R.E., Lin B., Maruyama T., McWhirter J., Yantiri-Wernimont F., Bowdish K.S., Xin H. (2008). Selection of anti-cancer antibodies from combinatorial libraries by whole-cell panning and stringent subtraction with human blood cells. J. Immunol. Methods.

[49] Veine D.M., Yao H., Stafford D.R., Fay K.S., Livant D.L. (2014). A D-amino acid containing peptide as a potent, noncovalent inhibitor of α5β1 integrin in human prostate cancer invasion and lung colonization. Clin. Exp. Metastasis.

[50] Salminen J.K., Tammela T.L., Auvinen A., Murtola T.J. (2016). Antiepileptic drugs with histone deacetylase inhibition activity and prostate cancer risk: A population-based case-control study. Cancer Causes Control.

[51] Kang H., Gillespie T.W., Goodman M., Brodie S.A., Brandes M., Ribeiro M., Ramalingam S.S., Shin D.M., Khuri F.R., Brandes J.C. (2014). Long-term use of valproic acid in US veterans is associated with a reduced risk of smoking-related cases of head and neck cancer. Cancer.

[52] Motawi T.K., Darwish H.A., Diab I., Helmy M.W., Noureldin M.H. (2018). Combinatorial strategy of epigenetic and hormonal therapies: A novel promising approach for treating advanced prostate cancer. Life Sci..

[53] Xu Q., Liu X., Zhu S., Hu X., Niu H., Zhang X., Zhu D., Nesa E.U., Tian K., Yuan H. (2018). Hyper-acetylation contributes to the sensitivity of chemo-resistant prostate cancer cells to histone deacetylase inhibitor Trichostatin A. J. Cell. Mol. Med..

[54] Ruscetti M., Dadashian E.L., Guo W., Quach B., Mulholland D.J., Park J.W., Tran L.M., Kobayashi N., Bianchi-Frias D., Xing Y. (2016). HDAC inhibition impedes epithelial-mesenchymal plasticity and suppresses metastatic, castration-resistant prostate cancer. Oncogene.

[55] Tsaur I., Hudak L., Makarević J., Juengel E., Mani J., Borgmann H., Gust K.M., Schilling D., Bartsch G., Nelson K. (2015). Intensified antineoplastic effect by combining an HDAC-inhibitor, an mTOR-inhibitor and low dosed interferon alpha in prostate cancer cells. J. Cell. Mol. Med..

[56] Shi X.Y., Ding W., Li T.Q., Zhang Y.X., Zhao S.C. (2017). Histone Deacetylase (HDAC) Inhibitor, Suberoylanilide Hydroxamic Acid (SAHA), Induces Apoptosis in Prostate Cancer Cell Lines via the Akt/FOXO3a Signaling Pathway. Med. Sci. Monit..

[57] Chen C.S., Wenig S.C., Tseng P.H., Lin H.P., Chen C.S. (2005). Histone acetylation-independent effect of histone deacetylase inhibitors on Akt through the reshuffling of protein phosphatase 1 complexes. J. Biol. Chem..

[58] Verheul H.M., Salumbides B., Van Erp K., Hammers H., Qian D.Z., Sanni T., Atadja P., Pili R. (2008). Combination strategy targeting the hypoxia inducible factor-1α with mammalian target of rapamycin and histone deacetylase inhibitors. Clin. Cancer Res..

[59] Ellis L., Ku S.Y., Ramakrishnan S., Lasorsa E., Azabdaftari G., Godoy A., Pili R. (2013). Combinatorial antitumor effect of HDAC and the PI3K-Akt-mTOR pathway inhibition in a Pten defecient model of prostate cancer. Oncotarget.

[60] Yan Y., An J., Yang Y., Wu D., Bai Y., Cao W., Ma L., Chen J., Yu Z., He Y. (2018). Dual inhibition of AKT-mTOR and AR signaling by targeting HDAC3 in PTEN- or SPOP-mutated prostate cancer. EMBO Mol. Med..

